# miR-216a Acts as a Negative Regulator of Breast Cancer by Modulating Stemness Properties and Tumor Microenvironment

**DOI:** 10.3390/ijms21072313

**Published:** 2020-03-27

**Authors:** Giuseppina Roscigno, Assunta Cirella, Alessandra Affinito, Cristina Quintavalle, Iolanda Scognamiglio, Francesco Palma, Francesco Ingenito, Silvia Nuzzo, Francesca De Micco, Antonio Cuccuru, Renato Thomas, Gerolama Condorelli

**Affiliations:** 1 Department of Molecular Medicine and Medical Biotechnology, “Federico II” University of Naples, 80131 Naples, Italy; giusy_roscigno@yahoo.it (G.R.); ass.cirella@outlook.com (A.C.); iolanda.scognamiglio@gmail.com (I.S.); 2 Percuros BV, 2333 CL Leiden, The Netherlands; aleaffi4@gmail.com (A.A.); cristinaquintavalle@gmail.com (C.Q.); frapalma9@gmail.com (F.P.); francesco.ingenito@outlook.it (F.I.); 3 IEOS (Istituto per l’Endocrinologia e l’Oncologia Sperimentale “G. Salvatore”), CNR (Consiglio Nazionale delle Ricerche), 80131 Naples, Italy; 4 IRCCS SDN (Istituto di Ricovero e Cura a Carattere Scientifico, SYNLAB istituto di Diagnostica Nucleare), 80143 Naples, Italy; nuzzo.silvia@gmail.com; 5 Mediterranea Cardiocentro, 80122 Naples, Italy; demiccofrancesca@hotmail.it (F.D.M.); amalianto@libero.it (A.C.); senologia@clinicamediterranea.it (R.T.)

**Keywords:** Breast cancer, cancer stem cells, microRNA, tumor microenvironment

## Abstract

Breast cancer is the most frequent malignancy in females in terms of both incidence and mortality. Underlying the high mortality rate is the presence of cancer stem cells, which divide indefinitely and are resistant to conventional chemotherapies, so causing tumor relapse. In the present study, we identify miR-216a-5p as a downregulated microRNA in breast cancer stem cells vs. the differentiated counterpart. We demonstrate that overexpression of miR-216a-5p impairs stemness markers, mammosphere formation, ALDH activity, and the level of *Toll-like receptor 4* (*TLR4*), which plays a significant role in breast cancer progression and metastasis by leading to the release of pro-inflammatory molecules, such as interleukin 6 (IL-6). Indeed, miR-216a regulates the crosstalk between cancer cells and the cells of the microenvironment, in particular cancer-associated fibroblasts (CAFs), through regulation of the TLR4/IL6 pathway. Thus, miR-216a has an important role in the regulation of stem phenotype, decreasing stem-like properties and affecting the cross-talk between cancer cells and the tumor microenvironment.

## 1. Introduction

Breast cancer is the leading cause of death among women: In fact, it represents 15% of all cancer deaths in females [1]. Normal adult stem cells are tissue-specific cells with particular features, such as the abilities to self-renew and to differentiate into all cell types of the tissue of origin. These cells undergo an unlimited number of cell divisions, and through symmetrical or asymmetrical division, they produce daughter cells that also have the ability to self-renew or differentiate, respectively. In a similar manner, cancer stem cells (CSCs)—which are resistant to conventional treatment and are implicated in tumor relapse and to the metastatic process—play a main role in the development and the progression of cancer [2,3]. Thus, a main challenge is to develop therapies that selectively target the CSC population. 

MicroRNAs (miRNAs) are small (20–23 nucleotide), endogenous, single-stranded RNA molecules involved in the post-transcriptional regulation of gene expression. Several miRNAs regulate genes involved in mechanisms such as proliferation, apoptosis, and stemness maintenance. Mutation in the biogenesis of these miRNAs can contribute to cancer development. Several miRNAs have been found to be involved in the regulation of the stemness phenotype [4]. For instance, let-7 is one of the main regulators of self-renewal in breast cancer cells [5]; miR-127 and miR-128 have been shown to be important in the modulation of stemness in glioma, since their ablation enhances stem cell renewal and proliferation [6]; and, miR-24 was proven to be a powerful positive regulator of breast CSC features during hypoxic conditions [7]. Moreover, miR-216a-5p—encoded on chromosome 2 on the reverse strand and highly conserved across species—has been shown to act as a tumor suppressor in gastric cancer, breast cancer, hepatocellular carcinoma, small cell lung cancer and renal cancer [8,9,10,11]. Little is known about the miR-216a regulation in cancer, but a recent manuscript by Tao W and colleagues reported that a long noncoding RNA named *DANCR* is able to target miR-216a-5p in breast cancer, regulating the expression of stemness markers such as *Nanog*, *SOX2* and *OCT4* [12]. 

The tumor microenvironment is largely involved in the regulation of neoplastic processes; it includes cells belonging to innate immunity, such as macrophages, neutrophils, and mast cells, and to adaptive immunity, such as T and B lymphocytes as well as stromal cells (fibroblasts, endothelial cells and mesenchymal cells) [13,14,15]. All these components of the tumor microenvironment interact with each other and cancer cells by either direct contact or through the release of cytokines and chemokines, which can act as paracrine or autocrine effectors [16,17,18]. The level of activation of these different cell types and the relative expression of the various mediators are able to tilt the balance in favor or against cancer progression [19,20]. 

Through modulation of gene expression related to the inflammation, miRNAs influence cancer-related inflammation, enhancing cancer tumorigenicity and aggressiveness. Cancer-associated fibroblasts (CAFs) are the main players in tumor stroma [21]. These cells release inflammatory cytokines, leading to the activation of pathways enhancing proliferation and stemness maintenance of cancer cells [22]. On the other hand, cancer cells release pro-inflammatory factors able to educate normal fibroblasts (NFs) into CAFs, generating positive feedback loops between cancer cells and CAFs [23]. A link between the microenvironment and cancer cells are the Toll-like receptors (TLRs), which have recently generated great interest in cancer research: these receptors are involved in the defense against microbial infection, but they can also support tumor cell growth in vitro and in vivo [24]. TLRs in general, and TRL4 in particular, play a significant role in breast cancer progression and metastasis by leading to the release of pro-inflammatory molecules [25]. Here we show that miR-216a regulates the stemness state by acting on TLR4. The up-regulation of this gene is implicated in stemness state regulation, controlling stemness pathways and interactions in the microenvironment.

## 2. Results

### 2.1. MiR-216a Negatively Regulates the Stemness Features and ADLH Activity of Breast Cancer Stem Cells (BCSCs)

**Figure 1:**
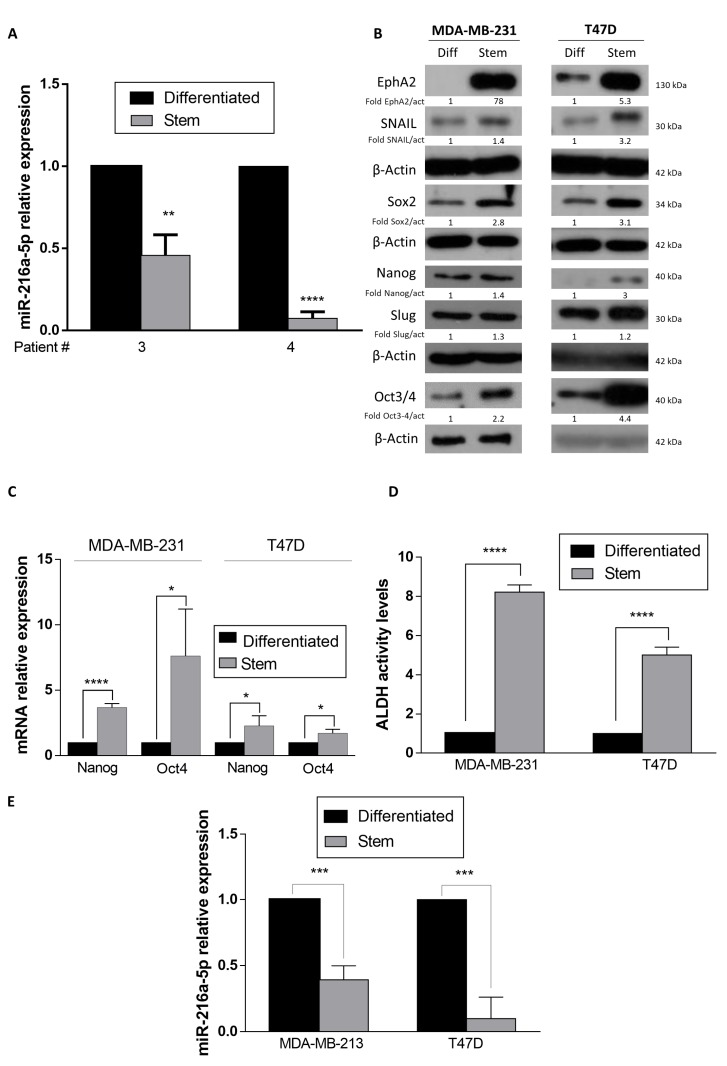
MiR-216a-5p expression in breast cancer stem cells (BCSCs). (**A**) MiR-216a expression levels were analyzed by qRT-PCR in primary breast cancer cells cultured in suspension (stem cells) or adherent (differentiated cells) conditions. (**B**) MDA-MB-231 and T47D stem cells express higher levels of stem markers compared to differentiated cells, as assessed by Western blotting. (**C**) MDA-MB-231 and T47D stem cells express high levels of *Oct4* and *Nanog*, as assessed by Real-time PCR. (**D**) MDA-MB-231 and T47D stem cells have high levels of ALDH activity, as assessed by flow cytometry. (**E**) MiR-216a is down-regulated in enriched stem cell cultures. Data are mean values ± SD of three independent experiments. Significance was calculated using Student’s t-test. *, *p* < 0.05, **, *p* < 0.01, ***, *p* < 0.001, ****, *p* < 0.0001.

To evaluate the role of miRNAs in breast cancer stemness maintenance, we previously performed a microarray analysis comparing miRNAs expression levels in differentiated vs. stem cells obtained from three patients’ specimens [26]. BCSCs were obtained by biopsy digestion and characterized by their ability to produce tumors when injected into immunocompromised mice and by real time PCR for the expression of the stem markers *Nanog* and *Oct4* [26,27]. The microarray results revealed there was a significant down-regulation of miR-216a [27]. We confirmed this finding by RT-PCR (Figure 1A). In order to obtain stem cell populations, we adopted suspension cultures of T47D and MDA-MB-231 breast cancer cells. In this condition, stem cells are enriched and grow as mammospheres. Through Western blot analysis and real-time PCR, we verified that the expression of stem cell markers such as EphA2, Snail, Sox2, NANOG, Slug and Oct3/4 was increased in cells grown as mammospheres compared to differentiated cells (Figure 1B,C) in both MDA-MB-231 and T47D cell lines. 

It has been reported that both normal and cancer stem cells express high levels of ALDH and that ALDH is a powerful predictor of poor clinical outcome as well as being a marker of stem/progenitor cells [28]. We compared ALDH activity levels in MDA-MB-231 and T47D stem and differentiated cells. We found that mammospheres had higher levels of ALDH activity (Figure 1D). Interestingly, miR-216a was down-regulated in the stem cell cultures in a similar manner to primary breast cancer cells (Figure 1E).

**Figure 2:**
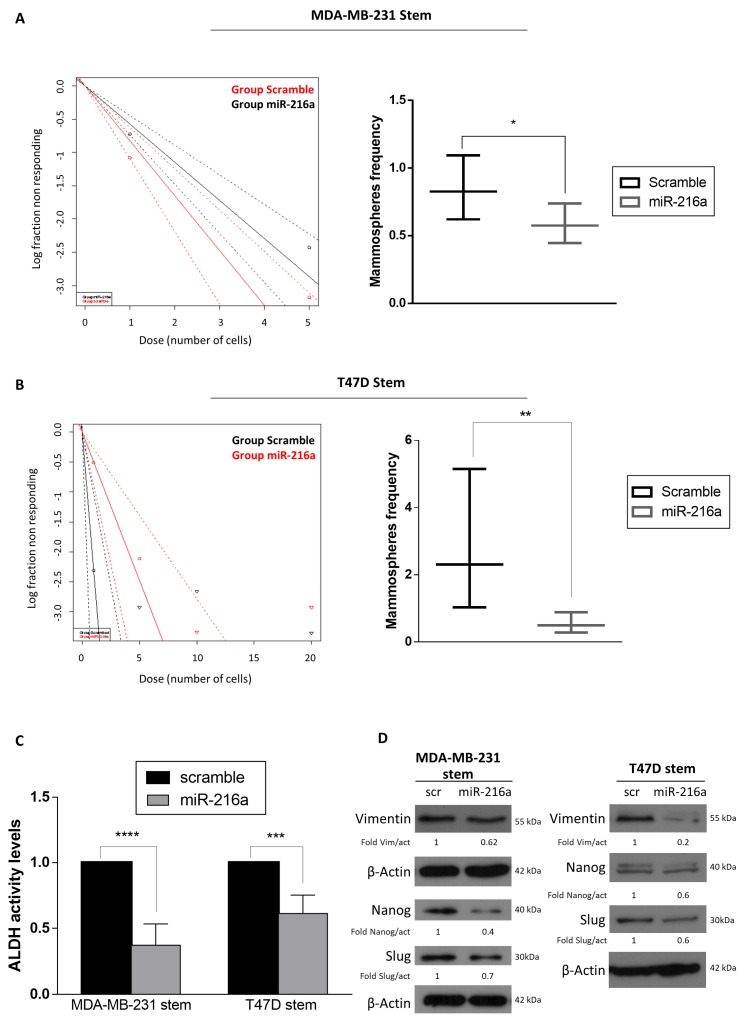
MiR-216a-5p regulates BCSC phenotype. (**A**,**B**) MiR-216a overexpression decreases the frequency of stem cells in MDA-MB-231 and T47D cultures, as assessed by limiting dilution assay. Significance was calculated using ELDA software. (**C**) ALDH activity decreases after transfection with miR-216a in MDA-MB-231 and T47D stem cells. (**D**) Stemness and EMT markers are decreased after transfection of miR-216, as assessed by Western blotting, in MDA-MB-231 and T47D stem cells. Data are mean values ± SD of three independent experiments. Significance was calculated using Student’s t-test. *, *p* ≤ 0.05; **, *p* < 0.01, ***, *p* < 0.001, ****, *p* < 0.0001.

**Figure 3:**
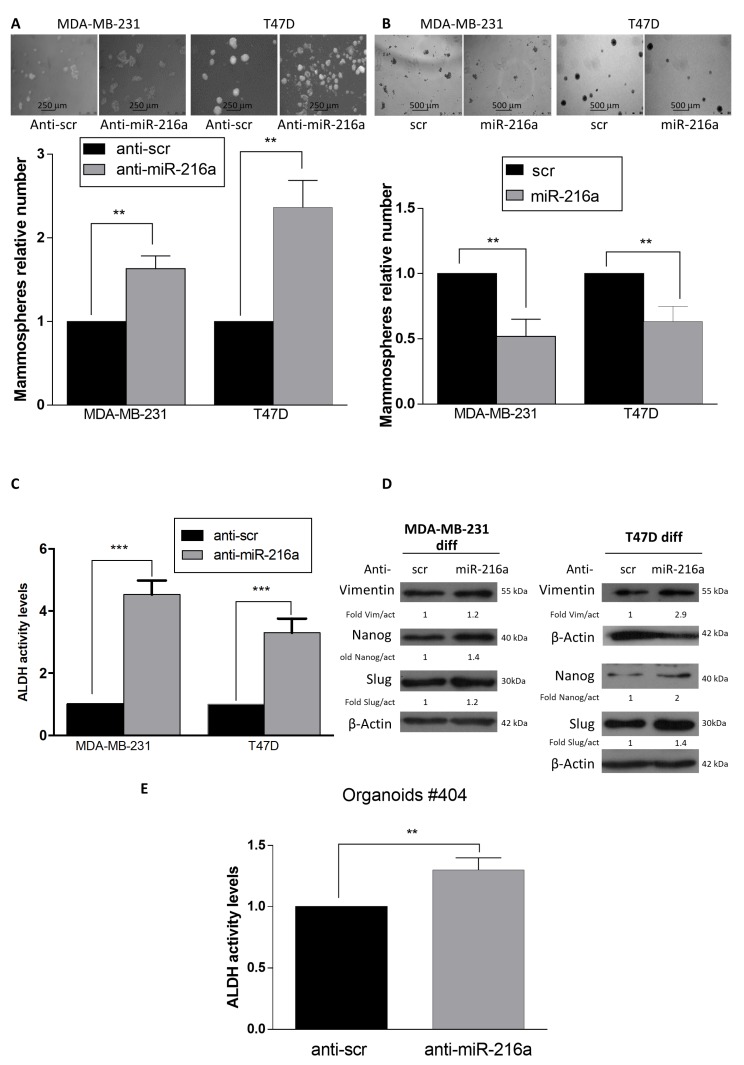
MiR-216a-5p increases stemness phenotype in differentiated breast cancer cells. (**A**) MiR-216a anti-sense oligonucleotide increases the number of mammospheres when transfected into differentiated MDA-MB-231 cells. Images were taken at 10x magnification, scale bar 250 µm. (**B**) MiR-216a overexpression decreases the number of stem cells in MDA-MB-231 and T47D cultures, as assessed by mammosphere assay. Images were taken at 5x magnification, scale bar 500 µm. (**C**) miR-216a downregulation, mediated by anti-miR-216a transfection, increases ALDH in MDA-MB-231 and T47D differentiated cell cultures. (**D**) Downregulation of miR-216a leads to a higher expression of stemness and EMT markers in differentiated MDA-MB-231 and T47D cells, as assessed by Western blotting. (**E**) ALDH activity increases after transfection with miR-216a anti-sense in patient-derived organoid cultures. Data are mean values ± SD of three independent experiments. Significance was calculated using Student’s t-test; **, *p* < 0.01, ***, *p* < 0.001.

In an effort to elucidate the role of miR-216a on stemness properties, we modulated miR-216 levels in stem cells and then performed a limiting dilution assay. The frequency of MDA-MB-231 and T47D stem cells in a mixed population decreased upon miR-216a overexpression (Figure 2A,B). Interestingly, miR-216a overexpression induced a decrease in ALDH activity in both MDA-MB-231 and T47D stem cells (Figure 2C, Supplementary Figure S1A). Additionally, overexpression of miR-216a decreased the expression of stemness markers (Nanog and Slug) and Epithelial Mesenchymal Transition (EMT) markers (Vimentin) in stem cells, as analyzed by Western blotting (Figure 2D). Conversely, downregulation of miR-216a in differentiated cells enhanced the capability of differentiated breast cells to form mammospheres, while overexpression decreased the propensity to become stem cells, as assessed by a mammosphere assay (Figure 3A,B, Supplementary Figure S1B). Moreover, down-regulation of miR-216a in differentiated T47D and MDA-MB-231 cells induced upregulation of ALDH activity (Figure 3C) and stem and EMT markers (Figure 3D). Moreover, to further confirm the role of miR-216a on stemness properties, we took advantage of an organoid culture system as a model closer to an in vivo experiment. For this, we transfected anti-miR-216 in breast cancer patient-derived organoids. Interestingly, we observed an increased ALDH activity (Figure 3E). Therefore, miR-216a negatively regulates stemness properties. 

### 2.2. miRNA-216a Targets TLR4

**Figure 4:**
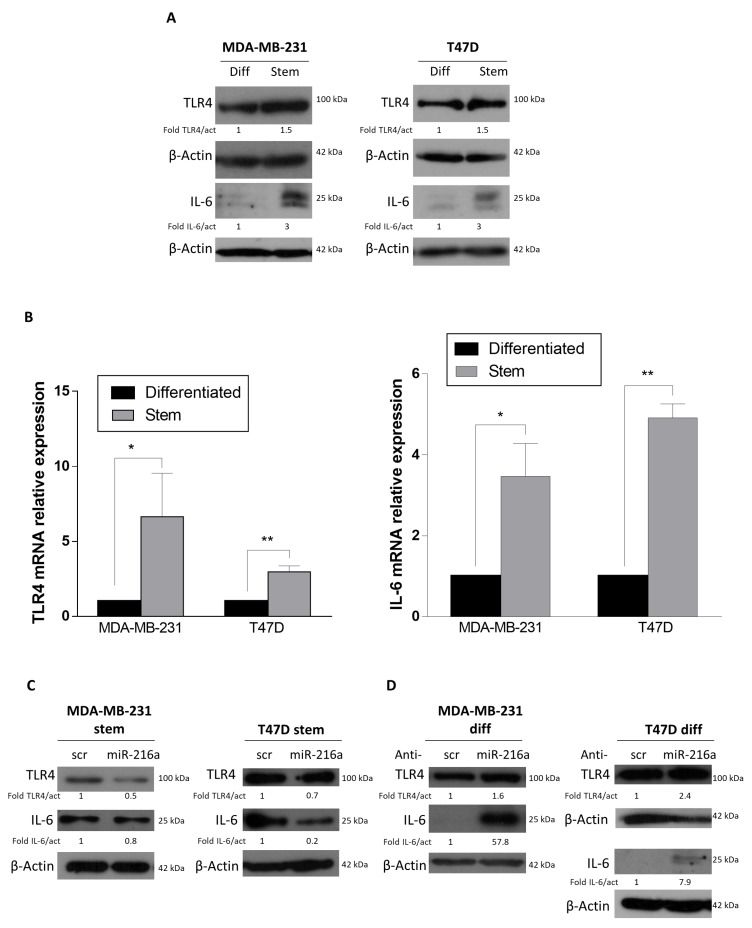
Toll-like receptor 4 (TLR4) is targeted by miR-216a-5p and regulates interleukin 6 (IL-6) levels. (**A**,**B**) TLR4 and IL6 protein and mRNA levels are higher in MDA-MB-231 and T47D stem cells compared to differentiated cells, as assessed by Western blotting and qRT-PCR, respectively. (**C**) MiR-216a overexpression induces a decrease in TLR4 and IL6 protein expression in MDA-MB-231 and T47D stem cells. (**D**) Conversely, by downregulating miR-216a, TLR4 and IL6 protein expression are increased. Significance was calculated using Student’s t-test. *, *p* < 0.05; **, *p* < 0.01. In (**C**) the blots representing TLR4 and IL-6 for MDA-MB-231 and T47D are from the same gel of Figure 2D. In (**D**) the blots representing TLR4 and IL-6 for MDA-MB-231 and TLR4 for T47D are from the same gel of Figure 3D.

We then investigated hypothetical miR-216a targets involved in the stemness phenotype. We used Target Scan Human as a bioinformatics tool for miRNA target prediction, focusing on a putative target, *TLR4,* which plays a significant role in breast cancer progression and metastasis [25,29] and that has also been reported to be a direct target of miR-216a-5p in renal carcinoma [30]. We found that *TLR4* expression was higher in MDA-MB-231 and T47D stem cells compared to differentiated cells at both protein and mRNA levels (Figure 4A,B). Interestingly, overexpression of miR-216a in stem cells decreased *TLR4* expression (Figure 4C). Conversely, expression of *TLR4* was enhanced by downregulating miR-216a in differentiated MDA-MB-231 and T47D cells (Figure 4D).

### 2.3. Role of miR-216a in Inflammation and Tumor Microenvironment Crosstalk 

Inflammation is a critical component of tumor progression [31]. Cancer cells release inflammatory cytokines and activate pathways that stimulate further cytokine production in CSCs. This generates a positive feedback loop driving CSC self-renewal and the activation of cells within the tumor microenvironment into tumor promoter cells. In particular, the interaction between cancer cells and fibroblasts results in the activation of the latter into CAFs [32]. Recent studies have demonstrated that *TLR4* expression and signaling induce an upregulation of IL-6 in different context such as colon cancer, Fragile X, and human bladder epithelial cells [33,34,35]. For this reason, we investigated whether miR-216a is involved in the crosstalk between cancer cells and the tumor microenvironment through the regulation of inflammatory cytokines IL-6. Interestingly, we found that IL-6 protein and mRNA levels were higher in MDA-MB-231 and T47D stem cells compared to their differentiated counterparts, due to higher levels of TLR4 (Figure 4A,B), and even more interestingly, *IL-6* expression levels changed upon TLR4 modulation induced by miR-216a (Figure 4C,D), confirming the crosstalk between TLR4, miR-216a, and IL-6. 

### 2.4. Role of miR-216a in the Tumor Microenvironment

**Figure 5:**
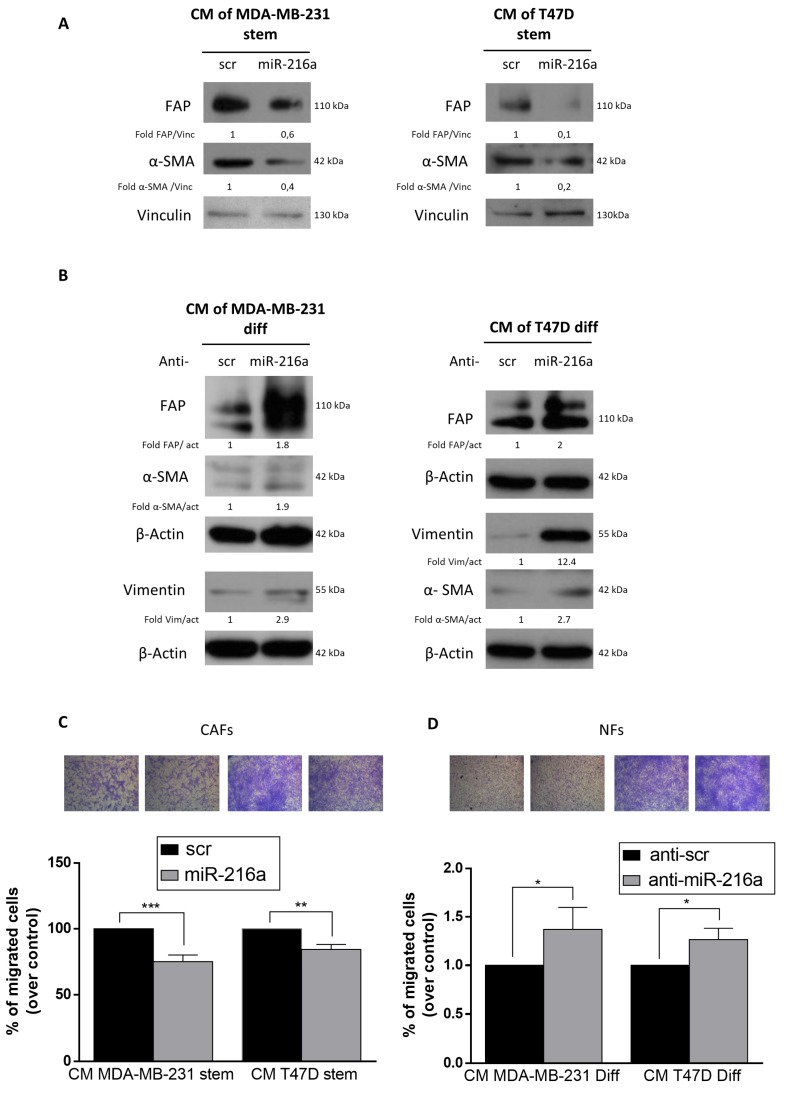
Effect of MiR-216a on cancer-associated fibroblasts (CAFs) and normal fibroblasts (NFs). (**A**) CAFs were treated with conditioned media collected from MDA-MB-231 and T47D stem cells overexpressing miR-216a. MiR-216a reduces CAF activation compared to the negative control. (**B**) Differentiated MDA-MB-231 and T47D cells transfected with anti-miR-216a upregulates CAF markers in NFs. (**C**) CAFs treated with conditioned media collected from MDA-MB-231 and T47D stem cells transfected with miR-216a. MiR-216a impaired fibroblast migration compared to the negative control. Images were taken at 5× magnification. (**D**) NFs treated with conditioned media collected from MDA-MB-231 and T47D differentiated cells transfected with anti-miR-216a. Anti-MiR-216a sequence induces migration of cells compared to the negative control. Images were taken at 5× magnification. Data are mean values ± SD of three independent experiments. Significance was calculated using Student’s t-test. *, *p* < 0.05; **, *p* < 0.01. ***, *p* < 0.001.

We next evaluated if downregulation of miR-216a in BCSCs induced fibroblast activation into CAFs. We treated CAFs with conditioned media collected from MDA-MB-231 and T47D stem cells transfected with miR-216a. After 48 h, cells were harvested and the expression of CAF activation markers (FAP and α-SMA) evaluated by Western blotting. We found that miR-216a-conditioned media reduced CAF activation compared to the control (Figure 5A). Conversely, the conditioned media from differentiated MDA-MB-231 and T47D cells upon downregulation of miR-216a induced education of NFs into CAFs (Figure 5B). In addition, CAFs treated with conditioned media collected from MDA-MB-231 and T47D stem cells transfected with miR-216a decrease their migration (Figure 5C), while conditioned media from differentiated MDA-MB-231 and T47D transfected with anti-miR-216a increased the migratory ability of normal fibroblasts (Figure 5D). Thus, in BCSCs, miR-216a decreases release of soluble factors that induce CAF activation, regulating the stem cell–microenvironment network.

## 3. Discussion

Cancer stem cells are supposed to be at the root of a tumor since they are able to self-renew indefinitely and generate mature cells [36]. Therefore, even if conventional therapy has improved life expectation, many patients still go through relapses because of the presence of CSCs in the tumor site [37]. Several studies have demonstrated that the expression of microRNAs is altered in the tumor setting [38], including in cancer stem cells [39], by modulating gene expression and, hence, stemness properties. Consequently, it appears evident that the study of microRNAs involved in the regulation of stemness features could provide valuable data for understanding the mechanisms involved in cancer progression and relapse. Here, we demonstrate that miR-216 acts as a stemness repressor in breast cancer stem cells, acting at different nodes. By microarray analysis performed on tumor patient specimens [26], we found that miR-216a-5p was downregulated in BCSCs compared to differentiated cells. Then, to elucidate the role of miR-216a in the regulation of the stem properties, we modulated the expression of the microRNA, upregulating it in breast cancer stem cells and downregulating it in differentiated cells. We verified that miR-216a-5p negatively regulates the number of stem cells, EMT, stem markers and ALDH activity. We then looked for possible targets, focusing our attention on TLR4, which belongs to a family of transmembrane receptors involved in the innate immune system [40]. TLRs protect the host against viral and bacterial infections and are able to trigger proinflammatory signaling cascades linking innate immunity to inflammation. TLR4 activation triggers the proinflammatory response, leading to the production and secretion of cytokines, including interleukin 6 (IL-6) [41,42] and IL-8 [43]. Several studies have been conducted to elucidate the links between TLR4 and breast cancer, and it has been shown that *TLR4* is highly expressed in breast cancer cells and plays a crucial role in invasiveness, migration and angiogenic potential of cancer at primary or metastatic sites [25,40]. In addition, *TLR4* knockdown reduces IL-6 and IL-8 secretion, cell survival and proliferation [44]. Through Western blot analysis, we found that this protein is regulated by miR-216, inducing us to consider it as possible axis involved in stemness features and microenvironmental cross talk. Interestingly, it has been already shown that miR-216a targets *TLR4* in renal cancer [30], corroborating our hypothesis. Since this protein is involved in inflammation pathways, we speculate that miR-216a has a role in the regulation of the inflammation network. Cancer cells interact with cells present within the tumor microenvironment, such as fibroblasts, immune and mesenchymal cells. It is known that breast CSCs modulate stromal cell activity (for example, transforming fibroblasts into cancer-associated fibroblasts) through cytokine secretion, leading them to release other cytokines that promote tumor malignancy. Moreover, in bladder cancer it has been reported that TLR4 signaling upregulates IL-6 in a dose- and time-dependent manner [34]. Conversely, *TLR4* knockdown reduces IL-6 and IL-8 secretion, cell survival and proliferation [44]. We demonstrate here that miR-216a-5p modulates *TLR4* to regulate the release of proinflammatory cytokines, such as IL-6, and that CAFs treated with miR-216-conditioned media express lower levels of CAF-activation markers compared to controls. 

In the present study, we explored the role of miR-216a in breast cancer, demonstrating that miR-216a acts as a negative regulator of stemness in BCSCs, influencing the expression of stem markers, their number and the interaction between BCSCs and cells within the tumor microenvironment. miR-216a regulates pathways involved in the control of stemness properties and cytokine production. Our finding shows for the first time that miR-216 modulates different aspects of cancer stem cells, regulating stemness pathways and microenvironment education. Our data demonstrate that miR-216 has pleiotropic effects and that it is downregulated in CSCs, exacerbating their malignant phenotype.

## 4. Materials and Methods 

### 4.1. Cells and Mammosphere Culture

Breast tumor differentiated cells from two patients (#3,#4) and BTSCs (Breast tumor stem cells) were obtained as previously described [26,45], and were used for the miR array. T47D (ER-positive) and MDA-MB-MB-231 (TNBC) differentiated cells were grown in RPMI 1640 medium supplemented with 10% heat-inactivated fetal bovine serum (FBS), 2 mM L-glutamine and 100 U/mL penicillin/streptomycin [46]. For mammosphere cultures, single cells were plated at a density of 1,000 cells/mL for T47D and 50,000/mL for MDA-MB-231. T47D and patients’ cells were grown in serum-free DMEM-F12 (Sigma, Milan, Italy), supplemented with B27 (Life technologies, Milan, Italy), 10 ng/mL EGF (Sigma), 20 ng/mL FGF (BD Biosciences, Milan, Italy) and 1X antibiotic-antimycotics (Life technologies). MDA-MB-231 stem cells were grown in Mammary Epithelial Cell Growth Medium (Lonza, Milan, Italy) supplemented with BPE, hEGF, insulin, hydrocortisone, GA-1000 (Lonza), B27 (Life technologies), and 20 ng/mL FGF (BD Biosciences, Milan, Italy). After 4–7 days, mammospheres, appearing as spheres of floating viable cells, were collected by gentle centrifugation (800 rpm) and dissociated with 0.25% trypsin for 5 min. 

### 4.2. Cell Transfection

For miRs transient transfection, cells at 50% confluency were transfected using Oligofectamine (Invitrogen, Life technologies) for differentiated cells, and Lipofectamine 3000 for stem cells (Invitrogen, Life technologies), with either 100 nM of pre-miR-216a-5p or scramble, or 200 nM of anti-miR-216a-5p and anti-scramble (Ambion, Life technologies).

### 4.3. Breast Primary Cell Culture

Breast carcinoma specimens were collected at the Surgical Unit of Clinica Mediterranea SPA (Naples, Italy). Prior consent was given by the donor before the collection, acquisition or use of human tissue. To obtain the cells, samples were mechanically and enzymatically disaggregated, and the lysates grown in DMEM-F12 medium supplemented with 10% FBS and 1% penicillin-streptomycin (Sigma-Aldrich). 

### 4.4. Organoids Cultures 

Tumor organoids were isolated using Hans Clevers protocol as reported before [47]. Organoids pellet was suspended in cold Cultex growth factor reduced BME type 2 (Trevigen, 3533-010-02) and 40 mL drops were allowed to solidify in a 24 multi-well suspension plates (Greiner, M9312) for 20 min at 37 °C. At the end, 400 μl of organoid medium were added for each well to cover the drop. Medium was replaced every 3–4 days. Organoids transfection was performed with Lipofectamine 3000 (ThermoFisher Scientific) according to manufacture instructions. 

### 4.5. Mammosphere Formation Assay

T47D and MDA-MB-231 cells were transfected with anti-miR-216a for 24 h. Mammospheres were generated according to the protocol published elsewhere [37,48,49]. Briefly, T47D and MDA-MB-231 differentiated cells were dissociated with 0.25% trypsin for 5 min. Then, cells were washed in serum-free DMEM-F12, centrifuged and the pellet was resuspended in stem media. 1000 or 5000 differentiated cells were counted and suspended in 2 mL of stem medium and finally plated into 6-well plates previously treated with polyHEMA to stop cell attachment. After 4–7 days, mammospheres, appeared as spheres of floating viable cells for T47D (ER-positive) cell line, while the TNBC cell line MDA-MB-231 appeared as bead-like structure. After 7/10 days for T47D and 10/15 days for MDA-MB-231, mammospheres were counted. Spheres of a diameter >50 μm for each well were counted under microscope. Representative images from mammospheres assay were used for each experiment [48,50].

### 4.6. Limiting Dilution Assay

After a 24-h transfection, 1, 5, or 10 cells were seeded per well of 96-well plates in stem cell medium. For MDA-MB-231 stem cells, we used polyHEMA-pretreated plates. Two weeks after seeding, the number of wells containing spheroids for each cell was counted. The analysis was performed using software available at http://bioinf.wehi.edu.au/software/elda13. We calculated the reciprocal of 95% confidence intervals for 1/(stem cell frequency) generated by ELDA software and the results shown in graph form as reported previously [51]. 

### 4.7. Conditioned Media

Media collected from stem cells cultures or differentiated cells were centrifuged at 1200 rpm for 5 min to remove cells and stored at −80 °C until used to treat fibroblasts for 48 h.

### 4.8. Flow Cytometry

The ALDEFLUOR assay was carried out according to the manufacturer’s guidelines (Stem Cell Technologies). Briefly, dissociated single cells were suspended in Aldefluor assay buffer containing an ALDH substrate, bodipy-aminoacetaldehyde (BAAA) (3 μL per milliliter of sample), and incubated for 40 min at 37 °C. A fraction of cells was incubated under identical conditions in the presence of an ALDH inhibitor (diethylaminobenzaldehyde (DEAB); 4 μL per milliliter of sample). Cells were analyzed using a BD Accuri C6 (BD Biosciences) flow cytometer with BD Accuri C6 software.

### 4.9. RNA Extraction and qRT-PCR 

Total RNA (miR and mRNA) was extracted using Trizol (Invitrogen, Milan, Italy) according to the manufacturer’s protocol. Reverse transcription of total miR was performed starting from equal amounts of total RNA/sample (500 ng) using SuperScript III Reverse Transcriptase (Invitrogen). Quantitative analysis of Zeb, Nanog, TLR4, IL-6, and β-actin (as an internal reference) were performed by Real Time PCR using specific primers and iQ SYBR Green Supermix (Bio-Rad, Milan, Italy). The reaction for detection of mRNAs was performed as follows: 95 °C for 15′, 40 cycles of 94 °C for 15′, 58 °C for 30′, and 72 °C for 30′. 

For miRNA expression analysis, 250 ng of the extracted RNA was reverse-transcribed using a TaqMan MicroRNA Reverse Transcription Kit. Quantitative analysis for microRNA-216a-5p and U6 (as an internal reference) were performed by qRT-PCR using specific Taqman probes.

All reactions were run in triplicate. The threshold cycle (CT) is defined as the fractional cycle number at which the fluorescence passes the fixed threshold. For relative quantization, the 2^−ΔΔCT^ method was used. Experiments were carried out in triplicate for each data point, and data analysis was performed by using Bio-Rad software.

### 4.10. Protein Isolation and Western Blotting 

Cells were washed twice in ice-cold PBS, and lysed with JS buffer (50 mM HEPES pH 7.5 containing 150 mMNaCl, 1% glycerol, 1% Triton X100, 1.5mM MgCl_2_, 5mM EGTA, 1 mM Na_3_VO_4_, and 1X protease inhibitor cocktail). Protein concentration was determined by the Bradford assay (BioRad) using bovine serum albumin as the standard, and equal amounts of proteins analyzed by SDS-PAGE (12.5% or 15% acrylamide). Gels were electroblotted onto nitrocellulose membranes (Millipore, Bedford, MA, USA), membranes then blocked for 1 h with 5% non-fat dry milk in Tris Buffered Saline (TBS) containing 0.1% Tween-20, and incubated at 4°C overnight with the primary antibody. Detection was performed by peroxidase-conjugated secondary antibodies using the enhanced chemiluminescence system (Thermo, Euroclone, Milan, Italy). Primary antibodies used were: anti E-Cadherin, anti-Nanog, anti-Slug, anti-Vimentin, anti-TLR4, anti-IL6, anti-FAP, anti-αSMA (Santa Cruz Biotechnologies, MA, USA), anti-β-actin (Sigma), and anti-vinculin (Cell Signaling). Western blots are from representative experiments.

### 4.11. Migration Assay

8.0 μm polycarbonate membrane permeable 6.5 mm transwell inserts (Corning Incorporated, NY, USA) were used to carry out the migration assay. Normal fibroblasts were harvested with a trypsin-EDTA solution (Sigma Aldrich, USA). 5 × 10^4^ cells were washed with PBS, then resuspended in 1% fetal bovine serum containing DMEM-F12 medium and seeded in the upper chamber. The lower chamber of the transwell was filled with 600 μL of culture medium containing 10% FBS. Cells were incubated at 37 °C for 24 h. The transwells were stained with 0.1% Crystal Violet in 25% methanol. Non-migrated cells were scraped off the top of the transwell with a cotton swab. The percentage of migrated cells was evaluated by eluting Crystal Violet with 1% SDS and reading the absorbance at λ 570 nm.

### 4.12. Statistical Analysis

Data are presented as mean ± standard deviation (SD). For comparisons between two groups, the Student’s t test was used to determine differences between mean values for normal distribution. All data were analyzed for significance using GraphPad Prism 6 software (San Diego, CA, USA). *p* values less than 0.05 were considered significant.

### 4.13. Ethics

The study was conducted according to the criteria set by the declaration of Helsinki and each subject signed an informed consent before participating to the study. The study was approved by the Research Ethics Committee of the University of Naples Federico II n° 119/15ES1.

## Supplementary Materials

Supplementary materials can be found at https://www.mdpi.com/1422-0067/21/7/2313/s1.

## Abbreviations

CSCs	Cancer Stem Cells
CAFs	Cancer Associated Fibroblasts
TLRs	Toll-Like Receptors
BCSCs	Breast Cancer Stem Cells
CAFs	Cancer Associated Fibroblasts
NFs	Normal Fibroblasts
