# Supplementary material for: miR-216a Acts as a Negative Regulator of Breast Cancer by Modulating Stemness Properties and Tumor Microenvironment
Source: Int J Mol Sci. 2020 Mar 27;21(7):2313. doi: 10.3390/ijms21072313 (PMC7178064; doi:10.3390/ijms21072313)
Supplement: Supplementary file 1 [file ijms-21-02313-s001.pdf]

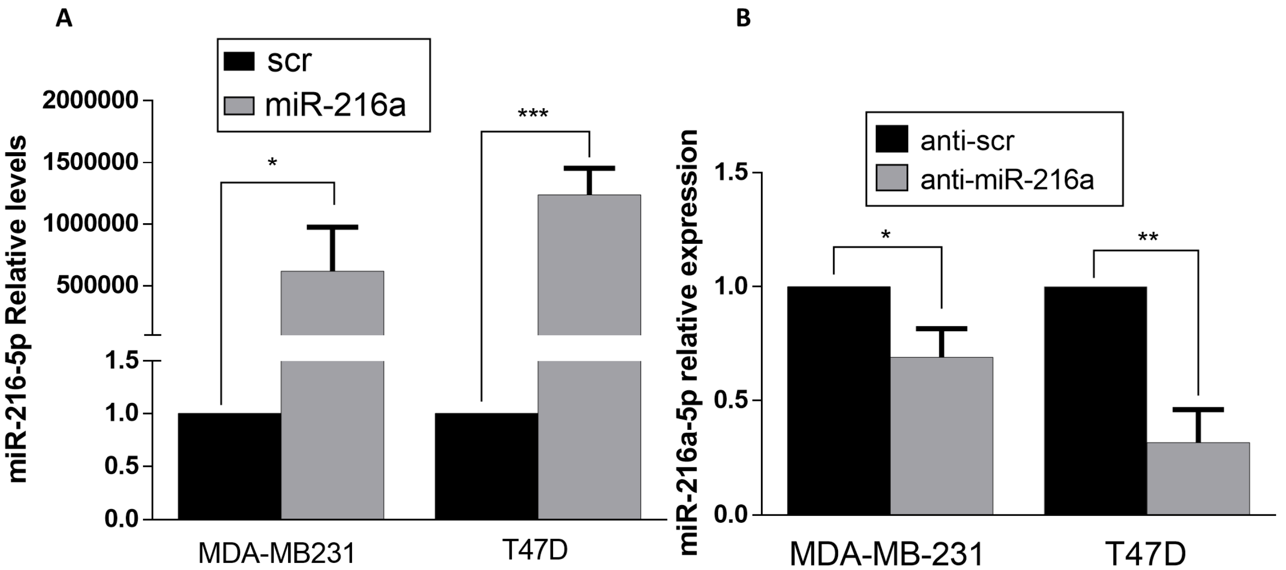

Supplementary Figure 1. MiR-216a expression levels after miR-216a pre-miRNA or anti-miRNA transient transfection. (**A**) qRT-PCR revealed the upregulation of miR-216a in breast mammospheres after miR-216a transfection with respect to scrambled control. (**B**) qRT-PCR revealed the down regulation levels of miR-216a in differentiated cells after anti-miR transfection with respect to scrambled control. Data are mean values ± SD of three independent experiments. Significance was calculated using Student's t-test .*, p<0.05; **, p<0.01. ***.
